# Novel missense variants in *LCAT* and *APOB* genes in an Italian kindred with familial lecithin:cholesterol acyltransferase deficiency and hypobetalipoproteinemia

**DOI:** 10.1016/j.jacl.2012.01.006

**Published:** 2012-05

**Authors:** Paola Conca, Silvana Pileggi, Sara Simonelli, Emanuela Boer, Giuliano Boscutti, Lucia Magnolo, Patrizia Tarugi, Silvana Penco, Guido Franceschini, Laura Calabresi, Monica Gomaraschi

**Affiliations:** a Center E. Grossi Paoletti, Department of Pharmacological Sciences, Università degli Studi di Milano, via Balzaretti 9, 20133 Milano, Italy; b Laboratory of Medical Genetic, Niguarda Cà Granda Hospital, Milano, Italy; c ASS2 “Isontina,”S. Giovanni di Dio Hospital, Gorizia, Italy; d Department of Biomedical Sciences, University of Modena and Reggio Emilia, Modena, Italy

**Keywords:** Apolipoprotein B, Cholesterol esterification, Familial hypobetalipoproteinemia, Familial LCAT deficiency, HDL subclasses, High-density lipoproteins, Lecithin:cholesterol acyltransferase

## Abstract

**Background:**

Lecithin:cholesterol acyltransferase (LCAT) is responsible for cholesterol esterification in plasma. Mutations of *LCAT* gene cause familial LCAT deficiency, a metabolic disorder characterized by hypoalphalipoproteinemia. Apolipoprotein B (apoB) is the main protein component of very-low-density lipoproteins and low-density lipoprotein (LDL). Mutations of *APOB* gene cause familial hypobetalipoproteinemia, a codominant disorder characterized by low plasma levels of LDL cholesterol and apoB.

**Objective:**

This was a genetic and biochemical analysis of an Italian kindred with hypobetalipoproteinemia whose proband presented with hypoalphalipoproteinemia and severe chronic kidney disease.

**Methods:**

Plasma lipids and apolipoproteins, cholesterol esterification, and high-density lipoprotein (HDL) subclass distribution were analyzed. *LCAT* and *APOB* genes were sequenced.

**Results:**

The proband had severe impairment of plasma cholesterol esterification and high preβ-HDL content. He was heterozygote for the novel LCAT P406L variant, as were two other family members. The proband’s wife and children presented with familial hypobetalipoproteinemia and were heterozygotes for the novel apoB H1401R variant. Cholesterol esterification rate of apoB H1401R carriers was reduced, likely attributable to the low amount of circulating LDL. After renal transplantation, proband’s lipid profile, HDL subclass distribution, and plasma cholesterol esterification were almost at normal levels, suggesting a mild contribution of the LCAT P406L variant to his pretransplantation severe hypoalphalipoproteinemia and impairment of plasma cholesterol esterification.

**Conclusion:**

LCAT P406L variant had a mild effect on lipid profile, HDL subclass distribution, and plasma cholesterol esterification. ApoB H1401R variant was identified as possible cause of familial hypobetalipoproteinemia and resulted in a reduction of cholesterol esterification rate.

Lecithin:cholesterol acyltransferase (LCAT) is a 416-amino acid protein responsible for the synthesis of cholesteryl esters in human plasma; LCAT is also one of the key enzymes involved in high-density lipoprotein (HDL) metabolism.^1^ The *LCAT* gene, located on chromosome 16q22.1, spans 4.2 kb and comprises 6 exons and 5 introns^2^; mutations in this gene cause LCAT deficiency, a rare metabolic disorder with two syndromes: classic familial LCAT deficiency (FLD; OMIM no. 245900), characterized by a complete lack of enzyme activity, and fish-eye disease (FED; OMIM no. 136120), characterized by a partially defective enzyme, both inherited via an autosomal-recessive patterns.^3^ Subjects with FLD and FED have hypoalphalipoproteinemia and impairment of cholesterol esterification in plasma.^3^ Homozygous FLD cases reportedly present with corneal opacity and anemia, and many of them unpredictably develop renal disease. The FED cases have a milder clinical phenotype. Heterozygous carriers have an intermediate lipid/lipoprotein phenotype, with a significant LCAT gene dose-dependent effect on plasma cholesterol esterification and lipid and apolipoprotein levels.^3^

Apolipoprotein B (apoB) is the major protein component of very-low-density (VLDL) and low-density lipoproteins (LDL) and plays a central role in human lipoprotein metabolism.^4^ The human *APOB* gene is located on chromosome 2p24-p23; it consists of 29 exons and produces, via a unique mRNA editing process, two forms of apoB: apoB-48 (2152 amino acids) and apoB-100 (4563 amino acids).^4^ Mutations in the *APOB* gene cause familial hypobetalipoproteinemia (FHBL; OMIM no. 107730), an autosomal codominant disorder characterized by very low plasma levels of total cholesterol, LDL cholesterol, and apoB.^5^ Most mutations interfere with the translation of full-length apoB mRNA and cause the production of truncated apoBs of various size; only few nonsynonymous, nontruncating mutations have been reported so far as cause of FHBL.^5–7^ Homozygous FHBL is extremely rare and it is associated with fatty liver, steatorrhea, intestinal fat malabsorption, and neurological abnormalities; FHBL heterozygotes may be asymptomatic or present with fatty liver disease and mild elevation of serum liver enzymes.^5^ In the present study, an Italian kindred with two novel missense variants in the *LCAT* and *APOB* genes is described.

## Methods

The study was approved by the local institutional ethic committee, and all subjects provided written informed consent for participation in the study.

## Subjects

**Figure 1:**
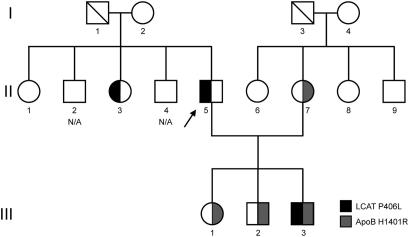
Pedigree of the family. The proband is indicated by an arrow. Left or right filled symbols indicate heterozygous carriers of LCAT (black) and apoB (gray) variants; white symbols indicate noncarrier relatives (square, male; circle, female). Slashed symbols indicate deceased individuals; N/A indicates family members not available for analysis.

The proband (subject II.5, Fig. 1), a 53-year-old white man from northern Italy, was referred to our lipid clinic for severe hypoalphalipoproteinemia (HDL cholesterol below the 5th percentile for the general population) during an epidemiological study on the dialytic population of an Italian Region in 2008. At 24 years of age, he had episodic macroscopic hematuria and demonstrated persistent microhematuria, proteinuria (1.5 g/L), and granulous cilindruria with normal renal function. Because of severe malignant hypertension and reduced renal function (creatinine clearance 58 mL/min), 6 years later he underwent renal biopsy that demonstrated chronic mesangial IgA glomerulonephritis together with severe hypertensive microvascular damage and no evidence of lipid deposits in the kidney.

Because of the following chronic renal insufficiency, he started dialysis therapy at 35 years of age. In 1990 he underwent a first renal transplantation from living donor (mother), but because of chronic rejection, he returned to hemodialysis in 2007. In 2009 he underwent a second, cadaveric, renal transplantation that is actually well functioning; he was given steroids, tacrolimus, and micofenolic acid (creatinine 1.7 mg/dL; creatinine clearance 57 mL/min). Blood samples were taken in 2008 (pretransplantation) and 10 months after the second transplantation (post-transplantation). The proband’s pedigree spans three generations and includes 14 living subjects (Fig. 1). All subjects had normal plasma creatinine and creatinine clearance values.

### Plasma lipids and apolipoproteins

After the patient fasted, his blood was collected into tubes containing Na_2_-EDTA (final concentration 1 mg/mL); plasma was prepared by low-speed centrifugation, and aliquots were immediately frozen at –80°C. Plasma total and unesterified cholesterol, high-density lipoprotein (HDL) cholesterol, and triglycerides were determined with standard enzymatic techniques. LDL cholesterol was calculated with the Friedewald’s formula. ApoA-I, apoA-II, and apoB levels were determined by immunoturbidimetry. The presence of lipoprotein X was evaluated by lipoprotein electrophoresis on agarose gels. ApoE phenotyping was performed by isoelectric focusing.^8^

### Genetic analysis

Genomic DNA was isolated from lymphocytes of peripheral blood samples using the salting out procedure.^9^ The six exons and the intronic flanking sequences of the *LCAT* gene were amplified by polymerase chain reaction (PCR) as previously described.^10^ PCR products were purified by solid-phase extraction (QIAquick PCR purification Kit; QIAGEN, Hilden, Germany) and sequenced with the Applied Biosystems Taq DyeDeoxy terminator cycle sequencing kit (Applied Biosystems, Carlsbad, CA). Sequencing reactions were separated on the ABI PRISM 3730 sequencer (Applied Biosystems).

The entire *APOB* gene-coding region, including the 5′-flanking region and at least 50 base pairs of intronic sequence at each intron–exon boundary, were amplified by PCR, as described previously.^11^ Sequences were detected on an Applied Biosystems 3100 DNA sequencer, and results were analyzed with ABI PRISM SeqScape software (Applied Biosystems).

All available family members and 100 healthy controls were screened for the novel variants in *LCAT* and *APOB* genes by denaturing high-performance liquid chromatography or by sequencing the appropriate *APOB* gene regions, respectively.

### In silico analysis of missense variants

A computational analysis of novel missense variants of LCAT and apoB was performed with the PolyPhen (http://genetics.bwh.harvard.edu/pph/) and PANTHER (http://www.pantherdb.org/) programs.^12,13^

### Cholesterol esterification process

The esterification of cholesterol within endogenous lipoproteins (cholesterol esterification rate), or incorporated into an exogenous standardized substrate (LCAT activity) was determined as previously described.^3^ Plasma LCAT concentration was measured by an immunoenzymatic assay.^14^

### HDL and LDL subclasses

The plasma concentration of HDL particles containing only apoA-I (LpA-I) and of particles containing both apoA-I and apoA-II (LpA-I:A-II) was determined by electroimmunodiffusion in agarose gel (Sebia Italia, Firenze, Italy).^15^ HDL subclass distribution for size was determined by nondenaturing polyacrylamide gradient gel electrophoresis of the d<1.21 g/mL plasma total lipoprotein fraction; the HDL profile was divided into three size intervals, small (diameter 7.2–8.2 nm), medium (diameter 8.2–8.8 nm), and large HDL (diameter 8.8–12.7 nm).^15^ HDL subclasses were also analyzed by nondenaturing two-dimensional (2D)-electrophoresis followed by immunodetection against human apoA-I and serum content of preβ-HDL was expressed as percentage of total apoA-I.^15^

LDL particle size was analyzed by nondenaturing polyacrylamide gradient gel electrophoresis with the Pharmacia Phast System (GE Healthcare BioSciences, Uppsala, Sweden), as previously reported.^8^

## Results

### Plasma lipids and apolipoproteins

Plasma levels of lipids and apolipoproteins are reported in Table 1. At the first evaluation in 2008, the proband (II.5) had very low levels of plasma HDL cholesterol and apoA-I and high plasma triglycerides; the unesterified/total cholesterol ratio was also higher than the normal reference value (<0.30). Biochemical analyses were extended to all available family members. No reductions of HDL cholesterol and apoA-I plasma levels were detected in proband’s relatives; only the proband’s daughter (III.1) had borderline high unesterified/total cholesterol ratio.

Interestingly, the lipid profile of the proband’s children (III.1, III.2, and III.3) and wife (II.7) was suggestive of FHBL, characterized by low plasma levels of LDL-cholesterol and apoB, with a vertical transmission of the lipid/apoB phenotype.^7^ Lipoprotein X was not detected in the plasma of the proband and of other family members. All subjects carried the E3/E3 phenotype.

### DNA sequencing analysis

The hypoalphalipoproteinemia associated with the high unesterified/total cholesterol ratio of the proband was suggestive of a defect in the cholesterol esterification process; thus, the analysis of *LCAT* gene was performed. The proband (II.5) was indeed found to be heterozygous for a novel point variant at c.1289 (C to T transition), which caused a leucine for proline substitution at position 406, P406L. Proband’s sister (II.3) and son (III.3) were also heterozygotes for the P406L variant. A cohort of 100 healthy controls was screened with denaturing high-performance liquid chromatography analysis to verify the frequency of the variant and no subject carrying the P406L substitution was found.

Because FHBL was also detected, the genetic analysis was extended to *APOB* gene. Subjects II.7, III.1, III.2, and III.3 were indeed found to be heterozygous for a novel point variant at c.4411 (A to G transition), which caused an arginine for histidine substitution at position 1401 (H1401R). The H1401R variant was not detected in 100 healthy controls screened by direct sequencing of the candidate gene region. The amino acid residue at position 1401 is highly conserved among species.

### In silico analysis of missense variants

The computational analyses performed with the PolyPhen and Panther programs suggest that the LCAT P406L variant should not affect LCAT function (PolyPhen prediction: benign; Panther SubPSEC -1.4115, P_deleterious_ 0.1696); on the contrary, the apoB H1401R variant is expected to have deleterious effect on apoB function (PolyPhen prediction: probably damaging; Panther SubPSEC -4.7431, P_deleterious_ 0.8511).

### Impact of variants on cholesterol esterification process

The proband (II.5) had markedly low cholesterol esterification rate, LCAT concentration, and activity (Table 2), a very severe biochemical phenotype for a heterozygous carrier of LCAT variants.^3^ Family members carriers of the LCAT P406L mutant or of the ApoB H1401R mutant showed reduced levels of plasma cholesterol esterification rate (Table 2). This impairment of cholesterol esterification was caused neither by reduced plasma levels of LCAT nor to a defective LCAT enzyme, since LCAT activity, which is measured on exogenous lipoproteins, was normal (Table 2).

### Impact of variants on HDL and LDL subclasses

The proband displayed a low plasma level of LpA-I:A-II and an increased percentage of preβ-HDL. On average, neither the LCAT P406L nor the apoB H1401R mutant significantly affected HDL subclass distribution for protein composition, size and shape (Table 3). Indeed, LpA-I and LpA-I:A-II levels were within the normal range; HDL distribution according to particle size and percentage of discoidal preβ-migrating HDL were also comparable between carriers of the mutants and non carrier relatives. No alteration of LDL size was detected (Table 3).

### Pre- and post-transplant values

**Figure 2:**
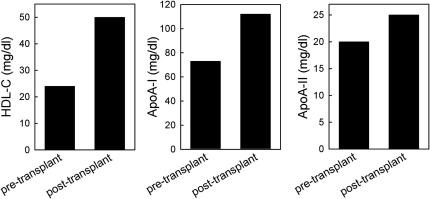
Pre- and post-transplantation lipid and apolipoprotein levels. Proband’s plasma levels of HDL-C, apoA-I, and apoA-II measured during hemodialysis (pre-transplant) and 10 months after the second renal transplant (post-transplant) are shown.

**Figure 3:**
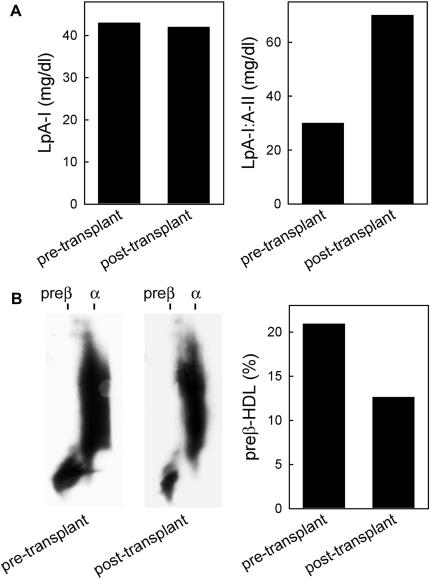
Pre- and post-transplantation HDL subclass distribution. A, Plasma levels of HDL containing only apoA-I (LpA-I) and HDL containing both apoA-I and apoA-II (LpA-I:A-II) measured during hemodialysis (pre-transplant) and 10 months after the second renal transplant (post-transplant). B, Representative 2D-electrophoresis developed against apoA-I and percentage of preβ-HDL by densitometry evaluated on proband’s plasma samples collected during hemodialysis (pre-transplant) and 10 months after the second renal transplantation (post-transplant).

**Figure 4:**
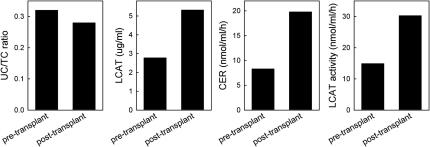
Pre- and post-transplantation plasma cholesterol esterification. Unesterified/total cholesterol ratio (UC/TC), plasma level of LCAT, cholesterol esterification rate (CER), and LCAT activity measured during hemodialysis (pre-transplant), and 10 months after the second renal transplant (post-transplant).

The proband’s lipid profile was reanalyzed 10 months after the second renal transplantation. Compared with values before the patient underwent transplantation, total and LDL cholesterol slightly increased to 227 and 144 mg/dL, respectively; triglycerides markedly decreased from 254 to 164 mg/dL. Notably, HDL cholesterol and apoA-I levels increased to 50 and 112 mg/dL, respectively (Fig. 2). HDL subclass distribution was also normalized: LpA-I:A-II increased from 30 to 70 mg/dL, whereas preβ-HDL decreased to 12.6% (Fig. 3). Plasma cholesterol esterification was also improved after transplantation; indeed, unesterified/total cholesterol ratio, LCAT concentration, and activity were normalized, whereas the cholesterol esterification rate increased but was still lower than reference values (19.8 nmol/mL/h; Fig. 4).

## Discussion

In the present work two novel missense variants in the *LCAT* and *APOB* genes are described. In a three-generation Italian kindred, three heterozygous carriers of the novel P406L variant of LCAT were found. This variant is localized within the proline-rich C-terminal portion on LCAT (residues 399–416); previous data showed that the complete deletion of this portion did not impair LCAT protein secretion, stability, and activity.^16,17^ In silico prediction of the impact of leucine for proline at position 406 of LCAT sequence indicated that this substitution between nonpolar amino acids should not affect LCAT expression and function. This prediction is strengthened by the biochemical analyses performed on LCAT P406L carriers. Subjects II.3 and III.3 did not display hypoalphalipoproteinemia and impaired cholesterol esterification in plasma except for a modest reduction of cholesterol esterification rate; this finding is in contrast with the average profile of Italian heterozygous carriers of LCAT mutations, which have unesterified/total cholesterol ratios greater than 0.30 and reduced LCAT activity.^3^

Further support to the mild effect of the LCAT P406L variant comes from the almost complete normalization of proband’s lipid profile 10 months after the second renal transplantation. Indeed, when analyzed in 2008 while being under hemodialysis, the proband displayed a severe reduction of plasma HDL cholesterol, LCAT concentration and activity, and cholesterol esterification rate that was suggestive of a very severe biochemical phenotype of LCAT deficiency; however, after renal transplantation, only cholesterol esterification rate was still below the normal range (as it was for carriers II.3 and III.3). Thus, the LCAT deficiency observed during hemodialysis was likely the result of the additive effects of the LCAT genotype and of the severe impairment of renal function.^18^ To further support this issue, renal biopsy showed chronic mesangial IgA glomerulonephritis and no evidence of lipid deposits in the kidney, a picture clearly different from the typical form of renal damage observed in subjects with genetic LCAT deficiency, that is focal glomerulosclerosis with vacuolization of glomerular basement membrane due to lipid deposition.^10^

According to the mild effect of the P406L variant on cholesterol esterification in plasma, HDL subclass distribution was not altered in the carriers; in particular, the percentage of preβ-HDL particles was not increased, as previously shown in Italian heterozygous carriers of LCAT mutations.^3^ In addition, proband’s preβ-HDL percentage, that was elevated under hemodialysis, was markedly reduced concomitantly with the normalization of cholesterol esterification achieved after renal transplantation.

Four family members presented with hypobetalipoproteinemia and were found to be carriers of a novel missense variant of apolipoprotein B. Mutations in the *APOB* gene causing FHBL are mainly frameshift or nonsense mutations leading to truncated apoB forms, whereas missense mutations are very rare.^5^ Here we found that subject II.7 and her three children (III.1, III.2, and III.3) are heterozygous carriers of a missense variant causing the substitution of the histidine at position 1401 with the other basic amino acid arginine. According to Segrest et al.^19^ this substitution is located within the amphipatic β1 domain of apoB, which generally is considered the major lipid-associating motif of the protein and involved in the initiation of triglyceride assembly in nascent apoB; on the contrary, the other missense mutations of apoB described to date are located in the N-terminal βα1 domain, likely involved in apoB intracellular trafficking and secretion.^7^

The in silico classification of the apoB H1401R variant as probably damaging associated with the vertical transmission of moderate hypobetalipoproteinemia in the kindred support apoB H1401R as possible causative of FHBL.^5^ However, in vitro expression of this novel apoB variant is needed to establish its biological effects. Carriers of the apoB H1401R displayed moderate reduction of cholesterol esterification rate; this reduction was not attributable to a reduced LCAT expression or defective enzyme activity, and it is likely consequent to the low amount of circulating LDL, which together with HDL, acts as LCAT substrates. Interestingly, subject III.3, who is a compound heterozygote for both the LCAT P406L and apoB H1401R variants, displayed a lipid profile, HDL subclass distribution, and plasma cholesterol esterification similar to that of his siblings III.1 and III.2, carrying only apoB H1401R, further supporting the mild effect of the LCAT P406L variant on carriers’ biochemical phenotype.

## Conclusion

The proband’s severe alteration of lipid profile, HDL subclass distribution and cholesterol esterification in plasma was the result of the LCAT P406L variant and of the concomitant chronic renal insufficiency. The novel apoB H1401R variant was found as possible cause of FHBL observed in the kindred and associated with a reduced cholesterol esterification rate.

## Financial disclosure

This work was supported in part by grants from Telethon-Italy (GGP07132 to L.C. and GGP08052 to M.G.) and from Fondazione Cassa di Risparmio di Modena (to P.T.).

## Figures and Tables

**Table 1:** Lipid and apolipoprotein levels

	I.2	I.4	II.1	II.3	II.5	II.6	II.7	II.8	II.9	III.1	III.2	III.3
Age, yr	89	75	68	63	54	48	47	42	39	18	14	12
Gender	F	F	F	F	M	F	F	F	M	F	M	M
LCAT genotype	WT	WT	WT	P406L	P406L	WT	WT	WT	WT	WT	WT	P406L
ApoB genotype	WT	WT	WT	WT	WT	WT	H1401R	WT	WT	H1401R	H1401R	H1401R
Total cholesterol, mg/dL	175	243	232	248	200	183	159	241	227	131	117	123
Triglycerides, mg/dL	76	94	91	81	254	54	58	102	85	72	44	41
Unesterified cholesterol, mg/dL	52	56	60	66	64	42	44	53	50	40	34	33
Unesterified/total cholesterol	0.29	0.23	0.26	0.27	0.32	0.23	0.28	0.22	0.22	0.31	0.29	0.27
LDL cholesterol, mg/dL	103	159	156	160	125	91	82	169	158	67	58	61
HDL cholesterol, mg/dL	57	65	58	72	24	81	65	52	52	50	50	54
Apolipoprotein A-I, mg/dL	120	153	131	165	73	175	129	131	124	103	91	109
Apolipoprotein A-II, mg/dL	23	31	33	33	21	25	25	28	25	22	26	31
Apolipoprotein B, mg/dL	77	124	112	117	88	69	58	132	117	50	40	45

**Table 2:** Cholesterol esterification process

	I.2	I.4	II.1	II.3	II.5	II.6	II.7	II.8	II.9	III.1	III.2	III.3	Reference values
LCAT genotype	WT	WT	WT	P406L	P406L	WT	WT	WT	WT	WT	WT	P406L	–
ApoB genotype	WT	WT	WT	WT	WT	WT	H1401R	WT	WT	H1401R	H1401R	H1401R	–
LCAT, μg/mL	5.60	5.44	5.97	5.53	2.78	4.49	5.23	4.94	4.85	4.99	5.45	5.60	3.1–6.7
CER, nmol/ml/h	38.2	47.3	42.1	24.1	8.3	29.4	21.1	39.9	34.3	23.0	19.8	19.3	30–60
LCAT activity, nmol/mL/h	37.9	41.9	38.3	37.1	14.9	45.2	25.2	38.5	37.6	33.7	45.2	35.5	25–55

ApoB, apolipoprotein B; CER, cholesterol esterification rate; LCAT, lecithin:cholesterol acyltransferase.

**Table 3:** HDL and LDL subclasses

	I.2	I.4	II.1	II.3	II.5	II.6	II.7	II.8	II.9	III.1	III.2	III.3	Reference values
LCAT genotype	WT	WT	WT	P406L	P406L	WT	WT	WT	WT	WT	WT	P406L	–
ApoB genotype	WT	WT	WT	WT	WT	WT	H1401R	WT	WT	H1401R	H1401R	H1401R	–
LpA-I, mg/dL	67	88	66	74	43	98	62	53	50	57	43	49	>42
LpA-I:A-II, mg/dL	53	65	65	91	30	77	67	78	74	46	48	60	>58
Small HDL, %	15.9	15.2	20.6	16.3	15.2	12.1	15.2	23.0	23.3	16.1	17.2	16.5	6.9–23.7
Medium HDL, %	11.3	19.5	17.3	14.2	18.1	22.2	14.9	23.0	21.6	11.4	12.2	17.1	13.5–30.3
Large HDL, %	72.8	65.3	62.1	69.4	66.7	65.7	69.9	54.0	55.1	72.5	70.6	66.4	50.5–75.3
Preβ-HDL, %	13.1	11.8	10.8	12.9	20.9	14.6	11.4	13.8	11.9	12.2	10.4	10.5	10–14
LDL size, nm	27.3	27.5	26.4	27.4	27.3	27.5	27.6	27.5	27.5	26.7	27.4	27.2	>25.5

HDL, high-density lipoprotein; LCAT, lecithin:cholesterol acyltransferase.; LDL, low-density lipoprotein; LpA-I, particles containing only apoA-I; LpA-I:A-II, particles containing both apoA-I and apoA-II.

Small HDL indicates HDL particle diameters <8.2 nm; medium, particle diameters 8.2 < nm < 8.8; and large, particle diameter >8.8 nm.
